# Operational Constraints and Gender Biases: A Qualitative Analysis of Physician Parenting Experiences

**DOI:** 10.1089/whr.2021.0099

**Published:** 2022-03-04

**Authors:** Hsin Lee, Heather L. Burrows, Kanakadurga Singer, Kirk J. Brower, Carol R. Bradford, Brooke Spencley, Lauren Owens, Helen Kang Morgan

**Affiliations:** ^1^ Department of Obstetrics and Gynecology, University of Michigan, Ann Arbor, Michigan, USA.; ^2^ Department of Pediatrics, University of Michigan, Ann Arbor, Michigan, USA.; ^3^ Department of Psychiatry, University of Michigan, Ann Arbor, Michigan, USA.; ^4^ Department of Otolaryngology-Head and Neck Surgery, University of Michigan, Ann Arbor, Michigan, USA.; ^5^ Obstetrics and Gynecology, University of Wisconsin, Madison, Wisconsin, USA.; ^6^ Department of Learning Health Sciences, University of Michigan, Ann Arbor, Michigan, USA.

**Keywords:** gender equity, female physicians, women in medicine, physician parents

## Abstract

**Objective:**

Although parenting responsibilities are correlated with gender disparities in professional development and salary, the nature of parental challenges is not well characterized. The aims of this study were to (1) illuminate faculty physicians' experiences with parenting and (2) identify system challenges and opportunities for improvement.

**Materials and Methods:**

In October 2019, a survey about parenting was sent to all physician faculty at a large Midwest academic medical center. Qualitative analysis of free-text response to the survey item “is there anything you wish to share about your experience of pregnancy or parenting as a physician” was performed. Themes were inductively identified and developed from the responses in a team-based iterative approach.

**Results:**

Of 2069 total physician faculty, 1085 (52.4%) responded to the survey and 253 (23%) of the respondents provided free-text comments. From these comments, the authors identified three themes as sources of challenges for physician parents: operational constraints, gender biases, and nontraditional or nonheteronormative family structures. Operational factors pertained to lack of scheduling flexibility, childcare challenges, lactation, colleague coverage, and transparency of policies. Responses indicated that gender biases are encountered by all genders, and expectations built on assumptions of “traditional” gender roles and family structure are problematic for many physician parents.

**Conclusion:**

Addressing the challenges and opportunities identified in the study is critical to building a more supportive institutional culture around parenting and to increase gender parity in academic medicine.

## Introduction

This is an important time for examining the professional factors that impact physician parents. Physician well-being and burnout have long been recognized as priority areas,^1,2^ with physician burnout rates reaching 40%–50%.^3^ Negative experiences with childcare responsibilities are associated with poorer indices of physician well-being.^4–6^ Over the past three decades, parenting considerations have become increasingly pertinent to physicians and their well-being. With the increase in women entering medicine and the workforce in general,^7–9^ physician fathers have taken on more childcare responsibilities.^10,11^ Nevertheless, physician mothers continue to take on the majority of childcare responsibilities within their family units.^10,12^

Furthermore, parenting responsibilities have been correlated with ongoing disparate professional development and salary between men and women.^13–17^ Physician mothers are more likely to turn down leadership and scholarship opportunities because of parenting responsibilities, yet are less likely to feel comfortable discussing parenting challenges with their leadership.^18^ It is also prescient to note that disruptions from the COVID-19 pandemic have led to unprecedented increases in challenges and stress for physician parents.^19^

We have largely gained our current knowledge of experiences in parenting as a physician through quantitative work, with many studies focusing on single specialties or primarily female respondents. The aims of our study were to (1) illuminate faculty physicians' parenting experiences at a large academic health system using qualitative analysis of free-text comments and (2) identify systems challenges and opportunities for improvement.

## Materials and Methods

We created a 31-item survey that asked respondents about gender, clinical department, parental status, perceptions of departmental support for pregnancy and parenting commitments, and perceptions of how pregnancy and parenting affect promotion processes. The primary analysis of the quantitative component has been described.^18^

The qualitative component was derived from a single item on the survey designed to elicit open-ended, unstructured, and brief narratives within a constructivist/interpretivist paradigm, by asking, “Is there anything you wish to share about your experience of pregnancy or parenting as a physician?” We elected this approach to understand the multifaceted experience as it is or has been lived and to minimize researcher influence on the responses given about their experience. The survey is available in Supplementary Appendix SA1.

We distributed the electronic Qualtrics survey by e-mail (Supplementary Appendix SA2) to all physician faculty at a large academic medical center in the Midwest in October 2019 with a 4-week data collection period. Reminder e-mails containing the link to the survey were sent once weekly during the data collection period.

Survey participation was voluntary and responses were captured automatically. We exported responses to the qualitative item on the survey in aggregate without demographic and practice identifiers for review and analysis. We also performed a secondary process in which responses were sorted by gender identity and exported without any other identifying factor. Respondents who included comments were compared to respondents who did not include comments through chi-square analysis of parental status and gender. Analysis of responses and creation of themes were guided by the goal of identifying (1) system challenges for physician parents and (2) opportunities for improvement regarding the physician experience.

Responses were independently reviewed and manually coded using standard thematic analysis techniques.^20^ Themes were inductively identified at a semantic level and developed from the responses (Table 1) using a team-based iterative approach by two physicians (H.K.M. and H.B.) with prior experience conducting qualitative analysis and one resident physician (H.L.). Two authors (H.K.M. and H.L.) independently coded the comments; after independent coding, the authors discussed themes and arbitrated differences. Validity was established through investigator triangulation, with themes independently confirmed by a third reviewer (H.L.B.). H.M. and H.L. reconciled final code/theme disparities.

**Table 1.** Demographics of Respondents Who Included Free-Text Comments

Characteristic	*n* (%)
Gender	
Male	96 (38)
Female	150 (59)
Other	1 (0)
No response	6 (2)
Parental status	
Parent	241 (95)
Nonparent	12 (5)
Specialty	
Procedural	79 (31)
Anesthesiology	20 (8)
Emergency medicine	15 (6)
Neurosurgery	0 (0)
Obstetrics and gynecology	8 (3)
Ophthalmology	9 (4)
Orthopedic surgery	2 (1)
Otolaryngology	7 (3)
General surgery	10 (4)
Surgical specialties	5 (2)
Urology	3 (1)
Nonprocedural	146 (58)
Dermatology	1 (0)
Family medicine	14 (6)
Internal medicine	67 (26)
Neurology	6 (2)
Pathology	7 (3)
Pediatrics	29 (11)
Physical medicine and rehabilitation	3 (1)
Psychiatry	6 (2)
Radiation oncology	2 (1)
Radiology	11 (4)
No response	28 (11)

This study was deemed exempt from regulation by the University of Michigan Institutional Review Board in August 2019 (HUM00159549). Informed consent was deemed not needed by the IRB exemption since responses were anonymously reported.

## Results

Of the 2069 total physician faculty, 1085 responded (52.4% response rate), with 955 (88%) of respondents identifying as parents. For the qualitative portion of the study, 253 (23%) of the respondents provided free-text, narrative comments. The demographics and characteristics of these 253 respondents are shown in Table 1. Respondents who included comments were more likely to be parents (*p* < 0.001) and women (*p* = 0.034) compared to respondents who did not include comments.

Responses to the free-text comments were rich in detail and length. The final data set included responses totaling 23,483 words; the average length was 51 words, with 42% of responses >50 words and 26% of responses >100 words. Many responses addressed multiple topics. Analysis of the comments revealed three overarching themes as sources of challenge: operational factors, gender biases, and nontraditional or nonheteronormative family structures. Table 2 describes the codes, subthemes, and how frequently they were included in the narratives.

**Table 2.** Codes, Subthemes, and Frequencies from Free-Text Comments

Code	Subtheme	Frequency
Operational factors		
Stress felt by parents being covered by colleagues	Colleague coverage	19
Nonparents feeling devalued relative to parenting colleagues		
Stress of covering colleagues due to parenting demands		
Leave policies are difficult to find and confusing, and there is a general lack of transparency	Leave policies	73
Leave policies are hard to implement/not implemented in accordance with policy		
Parental leaves are not sufficiently long for parenting needs		
Inability to alter or control operating room times conflicts with childcare needs	Work schedule flexibility	61
Inflexibility in making any change to clinic grids		
Meeting times tend to be at times (early morning and evening) conflicting with childcare responsibilities		
Time for lactation is absent or too short to facilitate lactation to the extent desired	Lactation	39
Lactation spaces are absent, sparse, not private enough, or too far to make them accessible		
Lactation support is generally absent/insufficient		
Balancing breastfeeding and pumping at work is stressful		
Desire to go part time, but could not due to financial concern	Finances	38
Arranging childcare is financially expensive		
The stresses of parenting are augmented when one is both the primary childcare provider and the primary source of income		
Went part time, despite financial concerns		
Particular stress/lack of options for finding emergency/unplanned childcare	Childcare	59
Lack of on-site childcare		
Lack of childcare available at times when physician families need it		
Insufficient access to high-quality childcare, including prolonged waitlists		
Gender biases		
Men are not as socially accepted or supported in their efforts to parent	Bias against men	17
Men are not given the same positive/desired accommodations to parent as women		
Respondents accredit the ability to be a physician parent to having a spouse who performs more childcare responsibilities	Gender assumptions and burden on partner	26
Spouses made professional sacrifices to perform more childcare responsibilities		
Leadership attitudes or policies assume that a physician has a spouse who performs childcare responsibilities		
Heterosexual physician couples particularly highlight gender differences in treatment/accommodations made for parenting for mothers compared to fathers	Two-physician households	14
One physician in a couple makes professional sacrifices to provide childcare among two-physician couples		
Challenges as a physician parent are augmented for those married to a physician		
Actions/performance being viewed differently due to one's parental status	Microaggressive comments	27
Subtly treated differently for the incorrect assumption of the need to parent		
Subtly treated differently and negatively for needing to parent		
Negative comments made to others and to themselves about parenting		
Women perform or are expected/assumed to perform the majority of childcare duties	Burden on women	37
Women disproportionately make professional sacrifices for childcare compared to men		
Current system disadvantages women professionally—“mommy tax”		
Men are perceived more favorably for performing the same childcare duties—“daddy bump”		
Older male leadership ignores/denies the role of gender bias in differential outcomes		
Importance of equal offering and utilization of paternity leave relative to maternity leave	Paternity leave	9
Nontraditional or nonheteronormative family structures		
The many additional and often unpredictable responsibilities of children with special needs	Special needs	8
LGBTQ parents have unique or additional challenges (e.g., need for fertility treatments) that are not commonly discussed	LGBTQ	1
Unique needs of adoptive parents	Adoptive parents	3
The many stressors of being a single parent	Single	9

LGBTQ, Lesbian, Gay, Bi-sexual, Transsexual or Queer.

### Operational factors

**FIG. 1.**
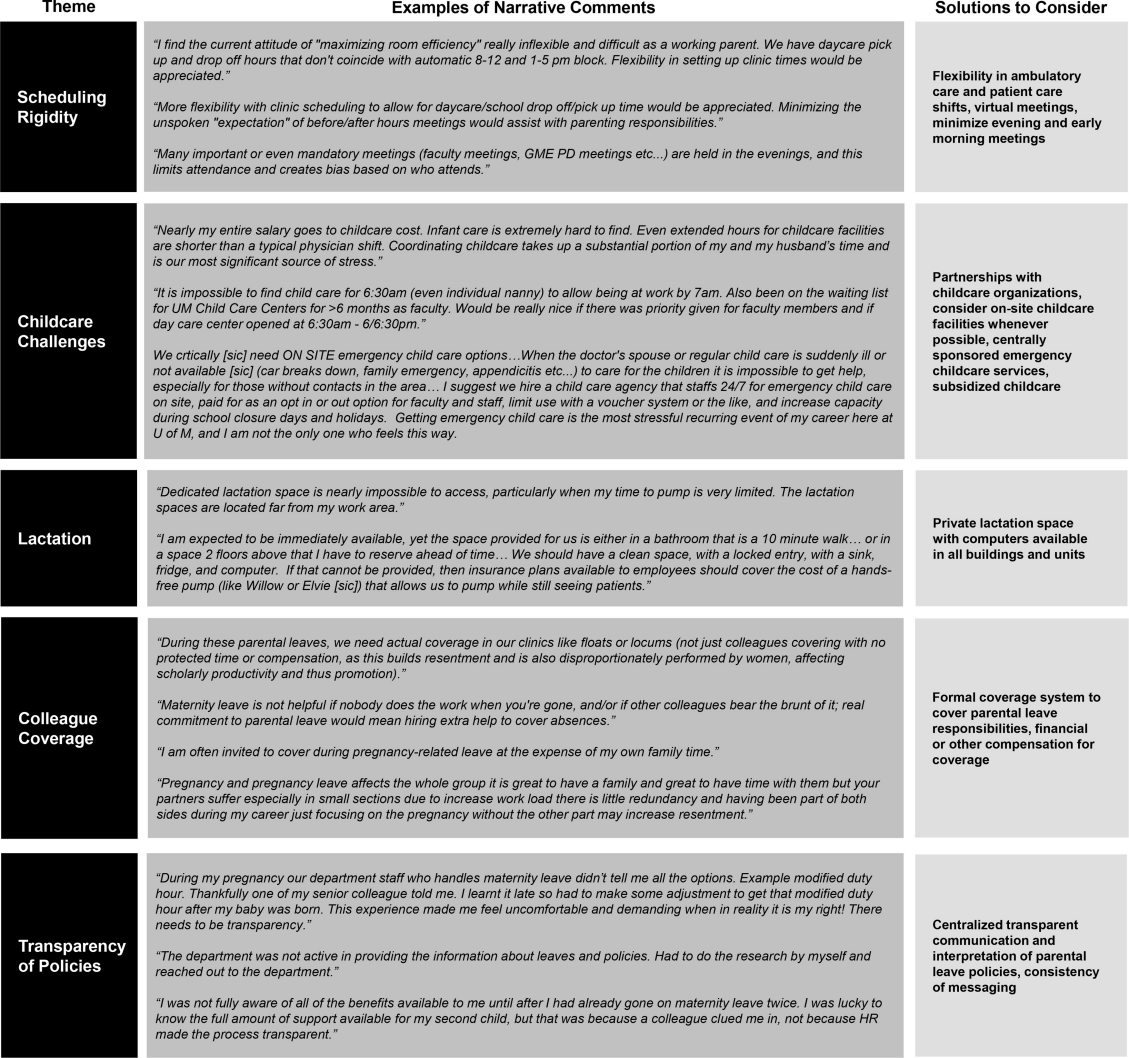
Operational constraints and possible solutions.

Many narrative comments pertained to operational factors that created challenges for physician parents, both at the time of pregnancy and parental leave, and later as children were older. Representative comments and proposed solutions are shown in Figure 1.

Lack of scheduling flexibility was frequently identified as an interference with childcare needs. Examples included institutionally mandated clinic grids that do not permit flexibility or individualization of work scheduling and evening or after-hour committee meetings, as respondents felt that their inability to attend committee meetings contributed to unequal career progression for physician parents. Several responses pertaining to scheduling flexibility emphasized the conflict between rigid schedules and unexpected or “emergency” parenting demands, such as when children are ill or school is cancelled unexpectedly. Frequently associated words in these responses were “scramble,” “stress,” “overwhelming,” and “no options.”

Securing available and accessible childcare was a very commonly expressed difficulty for several shared reasons. Respondents cited a lack of childcare options that align with the hours that physicians work, with earlier start times and later end times than usual day care hours, as well as the need to work night and weekend shifts. Lack of childcare options when unexpected or variable work demands arise, such as being called in to work overnight, was a further cited example of how existing childcare is not well fitted to physician work schedules. Difficulty in establishing one's family with a day care to begin with, due to very prolonged wait times compounded by the limited number of options, was a frequent occurrence and frustration that conveyed an overall childcare availability concern.

Many felt that space in the workplace and time in the workday for lactation were inadequate, impractical, or inaccessible. Respondents commented on too few lactation rooms, rooms with lack of privacy, insufficient time for pumping, and spaces located too far from where they were working to walk there, express breast milk, and return within the time permitted.

Colleague coverage was an area of concern. Parents and nonparent respondents identified the strain on a team resulting from the expectation that colleagues cover parents' work duties when they need to take care of their children.

Colleagues covering for parents expressed an understanding and willingness to cover for their colleagues, but that doing so is inevitably inconvenient, if not difficult, as physicians without children also have loved ones they desire time with—a sentiment anticipated by parents who expressed guilt over being a burden on colleagues. “Resentment,” “guilt,” and “unfair” were terms used in several responses addressing this current structure. A request was made in several responses for innovative and structured policies or processes to gain additional providers, such as float or locum providers, as opposed to informal arrangements to have existing providers gain additional duties.

Parental leave policy was the most frequently shared topic. Although sentiments regarding leave policies were a mix of positive and negative comments, they were unbalanced toward the negative; respondents expressed not only gratitude for improvement in policies over time but also abundant frustration toward current leave policy in relation to other countries, discrepancy between mothers and fathers, and overall time allotted for leave that is felt to be too short. Furthermore, the accessibility and utilization of leave policies were very commonly stated to be inadequate and inappropriate.

Many respondents shared difficulties with a lack of transparency of institutional or departmental policies pertinent to parenting. Difficulty in clarifying or utilizing parental leave policies was a common experience. Respondents felt administration and/or department leadership was not forthcoming with information regarding parental leave, with some sharing experiences of finding information about leave policies through informal word of mouth, inefficient and/or inaccurate collection of information, or not at all. Some respondents missed their eligible paid parental leave due to delays in processing or lack of awareness of policies in place until they were no longer eligible.

### Gender biases

Respondents described the expectation that women physicians fulfill the entirety or majority of parenting duties, and that this is a major barrier to promotion and professional growth for women. They felt this disproportionate expectation contributed to stalled career progression, inability to participate in research or committee meetings, not being offered comparable retention packages, and overall ongoing inequity in the physician workplace relative to male colleagues. Responses described compromised professional growth for women who served as the primary caregiver for their children, as well as for women who did not, but were assumed to. The overall sentiment was that the current system disadvantages women relative to men, with parenting expectations and duties being a direct mediator.

Importantly, very few responses focused on aspects of being a parent that are innate to being biologically of female sex, such as the experience of being pregnant, breastfeeding, or giving birth. Instead, they focused on cultural expectations and societal roles that women are expected to fulfill and described how these conflict with the expectations of being a physician—especially when the structure of physician responsibilities could be modified to be more manageable for parents, but have not yet been improved, as shared above in operational factors. This suggests that the sense of injustice conveyed in the responses is augmented by the sentiment that not only does the current inequality that disadvantages women exist but also that it does not inherently have to be the case.

“But physician mothers suffer a double edged stigma of being either not serious about their careers or being bad mothers. Some of it also is the societal expectation that women are traditionally the caregivers so the burdens of childcare disproportionately fall on women. This includes physician mothers who want to have careers and may or may not also have a working or supportive spouse. Often it is the woman physician in a man-woman partnership who has to cut down on her work commitments and make professional sacrifices to provide childcare.

Being able to go down to part time is not a victory if women have to choose between having a healthy family or their own sense of professional fulfillment and accomplishment. However societal expectations of women also includes [sic] women physicians who don't have children but are expected to perform a disproportionate share of other nurturing roles even in the professional setting (e.g., education, coverage for parent colleagues, etc.)”

“Turning down professional opportunities as a result of parenting is normal - it is work/life balance as they say. however, this falls disproportionately on women in our society, resulting in disparities at work. as a male, I am lucky. minimal parenting gets me kudos. for my wife (also a physician), society expects her to do it all.”

“Maternity leave should come out of both clinical and research time. Often, our clinical obligations are not “pro-rated” when we go on leave; everything comes out of research time. Yet promotions are based upon research productivity, so that works against women faculty.”

“I have reduced my faculty appointment, not gone to national meetings, cut patients off if they run overtime at the end of the day, decreased the quality of my chart notes, taken over a month to get patient's lab results to them, and more all to be able to continue society by raising a family. My division head and clinic head are both considered “nice guys," but deny that my problems are sex-specific and think that them [sic] picking up their kids a couple times a week means that they understand the position of working physician mothers. My division head expressly told me that my concerns ‘are not a sex issue and I don't want it brought up again.’ The fact is, every social survey shows that women do more housework and childcare than men do, even if the women are working as many hours as the man, yet there is an institutional blindness in the men here.”

Respondents described microaggressive and derogatory comments diminishing the importance of parenting as part of the work experience of women's efforts to parent. The sentiment behind the comments was most commonly that time away from work to parent is leisure time, which respondents felt to be insulting, as this time was actually spent performing necessary childcare. Terms used to describe what it felt like to receive these comments included “shame,” “guilt,” “stigma,” “unwelcome,” “inappropriate,” and “hostile.”

Respondents shared a sense of “pressure” to parent less as a result of disparaging comments. Furthermore, parent respondents described a cynicism that inclusive parenting policies served only as “lip service,” in that policies may seem supportive of parenting, but casual conversations in which microaggressive comments would be made would then reflect a reality of an unwelcoming attitude toward parenting.

“Can we not have men look down at us for being mother [sic]. I have worked part time and one had [sic] our division chair ask – ‘what do you do on your days off’[sic].”

“My experience was before modified duties and LOA policies. I was told to ‘enjoy my vacation.’ [sic] When I took breaks to pump, I was told I was slowing down clinic and couldn't I wait until lunch. I have no lunch, there is always a noon meeting. I pumped twice per day at best and felt shamed.”

Negative biases against men were related to assumptions that women should parent more than men, demonstrating that gender biases negatively impact both men and women. Men described feeling judged for dedicating or prioritizing time not at work to parent, as well as not being permitted time away from work to parent. Respondents identified formal and comparable parental leave for both male and female physicians as a necessity in improving the workplace experience. Respondents described a desire for paternity leave to be as equally utilized as maternity leave and how a lack of paternity leave perpetuates the gender biases with parenting.

“As a male parent with part-time homemaking responsibilities, I feel my division and department are highly biased against me compared to mothers in similar circumstances and I am not given the work hour accommodations and flexibility that are afforded mothers in identical circumstances. There is clearly a gender bias regarding the role of fathers in parenting responsibilities and I feel very disrespected by my supervisors for taking on parental responsibilities. The DEI movement here seems to look after women and mothers in terms of promotion, tenure, work-life balance, etc. but ignores the same bias against fathers.”

“I applaud the accommodations given to women with children. I think the same accommodations should be given to men with children. Otherwise, we are implicitly promoting the notion that childcare is women's work and men should not be sharing equally in the raising of their children. My wife and I both work for the University, but because of unequal rules in terms of maternity/paternity leave, tenure clock, etc. [sic], we felt pressure to have her take on more childcare responsibilities than me regardless of what our personal preferences were as a couple.”

“Male physicians should also take parental leave. When they choose not to utilize their benefits it makes a statement that they are not vital to providing care to their spouse and child. And continues to perpetuate a system that is disadvantaged for working women.”

Many respondents accredited the ability to be a parent and a physician to having a spouse—specifically and most commonly a wife—who took on the primary caregiver role. These wives consequently sacrificed their professional trajectories to make parenting work as a family unit. Female respondents who were part of a two-physician couple reiterated this gendered sacrifice in their responses as well, which is further described in the theme below.

Female physicians also described frustration that some department leaders seemingly had unrealistic expectations by failing to appreciate that they may not have the same support to balance work and parenting as physician fathers historically did. This was especially true among the physician mothers, who were more likely to be both the “breadwinner” as a physician, yet still had many childcare responsibilities.

“I have turned down some opportunities (papers, grant participation) but mostly I work 60 to 70 hours per week and get it done. The problem has been that my work demands have had substantial impact on my wife. She has had to bear the brunt of childcare activities, for the two of us, and this has significantly limited her career opportunities. She worked part time for years, then full time but in a position for which the expectation was 40 hours per week. Now that our kids are ready to leave home soon, she is left at middle age feeling like her career has not progressed anywhere, and her options at this later point in her career are limited.”

“The decision of my wife and I was to prioritize our children to the degree that she gave up her career to be a full-time caregiver early in their life. The cost of this far exceeds any childcare that reasonable people would afford.”

“I have been told by older male attendings that they don't understand why we complain so much as they did it with kids … ” “I [sic] want to point out they had a stay at home wife and I AM the stay at home wife and the breadwinner!”

Respondents described the challenges faced by physician parents as being amplified for families in which both parents are physicians. Respondents from two-physician households compared attitudes, experiences, and outcomes between their respective departments and between being a father versus being a mother—particularly highlighting the impact of gender on how one is treated as a parent. Reflections from two-physician households were congruent with other themes, such as maternity leave being more accessible, one partner making sacrifices to their career to care for children so the other partner can continue to focus on professional success, and frustration and difficulty with scheduling based on assumptions of having a spouse who is available to provide childcare.

“We are a two physician family. I have felt I needed to give up speed of promotion/%FTE to care for our kids in order [sic] that my spouse could advance more quickly.”

“I am constantly asked to attend evening meetings which is problematic as I am the primary care giver in a two physician family. We already have two nannies who work for us to cover all the hours we work. At least once a week I need to ask the nannies to stay late to attend meetings, and this is with turning down many evening meetings. All of the senior members of my division are men aged 60 and older who's [sic] wives stayed home to raise their children, so they do not understand how difficult it is to regularly hold meetings after 5pm.”

“I have been fortunate to have supportive leadership in my department when a childcare situation occasionally outstrips my various backup plans, but nonetheless it has been very clear that I have this degree of sympathy because I am a mom - my husband did not have the same courtesy extended. As a mom & doc, I often hear compliments about how I balance all of this; dad docs are assumed to have partners who can provide childcare and leave work if there's a problem. So, I'm part-time, and my partner is full-time; I'm a worker bee and he's in a leadership role; this seems like both reflection and confirmation of the expectations we faced.”

### Nontraditional or nonheteronormative family structures

Parents of children with special needs described increased demands and difficulties. They shared that their unique/additional needs were frequently absent from dialog in hospital workplaces when it comes to parenting, adding an additional challenge to the experience of physician parenting.

“I have a child with special needs that has required a lot of care. The burdens associated with children with special needs has not been addressed at all and has been entirely relied on my division's good will.”

“As a faculty member, though I do not anticipate having more children, I am struggling with a new diagnosis of autism in an older child, and fear that the additional time I need to care for him will affect my ability to be promoted on time. Ideally, there should be some accommodation for both new parents and for those parenting special needs children (who may be older) to help them meet professional goals.”

Workplace policies and structures were described as assuming that one is in a heterosexual relationship with biological children if one desires to parent, leaving LGBT, adoptive, and single parents feeling particularly unsupported. The lengthy time requirements and significant financial cost of assisted reproductive technology and adopting were specific strains of parenting efforts more commonly felt by LGBT, single, and adopting providers, with these considerations described as forgotten or missing from current dialog regarding parenting.

“The conversations focus on cis-heterosexual couples. Single, adopting, and same-sex/queer families are very marginalized and invisible. In particular, lots of subtle or overt discriminatory policies, despite people's best intentions. Reproductive services are completely cis-heteronormative and place multiple barriers on people who aren't cisgender heterosexual couples, with little facilitation, on same-sex/queer/single parents.”

“The time and expense of considering adoption has been very challenging. I have felt like there is less support for residents and faculty who are trying to adopt vs. those who are pregnant.”

## Discussion

This thematic analysis of free-text comments from our comprehensive survey of physician faculty demonstrates key challenges pertaining to operational constraints and gender biases related to parenting. While work-family conflict has been described and its impact on physician well-being established, our study contributes valuable details regarding the specific challenges related to institutional culture and systemic challenges that have not been as well described.

Responses in our study are consistent with three major themes in the existing literature: (1) organizational policies and practices assume a stay-at-home spouse,^21^ (2) flexible work schedules are perceived as beneficial for work-life balance,^21,22^ and (3) gendered societal expectations have a significant impact on the disparate challenges, sacrifices, and trajectories of physician mothers.^4,21,23,24^ In addition, similar operational constraints, such as meeting times and lack of emergency childcare, were identified decades ago as challenges faced by female faculty that affected work productivity and career satisfaction.^16^ Our study demonstrates that these systems barriers remain significant stressors for physician parents.

This work adds to existing literature by highlighting how parenting considerations and stresses impact both male and female physicians, with 40% of respondents who included comments being male. One important and novel theme that emerged in our analysis was discrimination and bias directed at male physicians regarding parenting considerations. To achieve gender equity, traditional patriarchal assumptions of gender roles need to be dismantled—for all genders. This work also highlights important equity considerations in terms of nonheteronormative family structures, with the unique challenges of single parenthood, Lesbian, Gay, Bisexual, Transsexual or Queer fertility demands, and children with special needs.

Much of the existing literature regarding work-family conflict and establishing the role of parenting in this conflict is quantitative. In this qualitative study, our respondents contribute depth, nuance, and detail to the understanding of *how* the various factors relate to the experience of physician parenting and better point to concrete measures that could be implemented to improve culture and overcome systemic challenges.

The narrative comments from our survey point to actionable measures that could be implemented to improve culture, which furthers the work of existing qualitative work regarding work-family conflict. Strong et al. found in their narrative responses that institutional culture and stigma seem to limit the utilization and potential benefit to physician satisfaction with work-life balance that is offered by family-friendly policies.^21^ The size of our study and the richness of the responses permitted more detailed insight into specific examples of experiences encountered by physician parents, which create a perceived culture that limits utilization, even when policies may offer the potential for a more family-friendly work schedule.

One example is increased transparency around pregnancy and parental leave policies that could be readily implemented at institutional and departmental levels. Given how common pregnancy and parental leave are for physicians, transparent and readily accessible policies will improve access and utilization, decrease burden for all stakeholders, and improve the perceived culture regarding parenting. The descriptions of microaggressive interactions and negative remarks shared by our study participants also highlight specific aspects of the culture around physician parenting that can undermine well-intended improvement offered by policy changes that are not fully utilized due to culture and stigma.

One of the most commonly included requests among our respondents was for flexibility with clinic scheduling and committee meetings—a suggestion also echoed by Lee et al. in their work evaluating work-life balance and conflict among medical faculty.^25^ While Lee et al. collected responses until saturation was met, our study welcomed reiteration of themes, with frequency of mention perhaps strengthening how widely felt or how important these work factors may be to physicians when it comes to better optimizing work-life balance. Measures for improvement such as flexibility with clinic scheduling grids and committee meeting times will require leadership buy-in, which will be challenging given the current financial challenges resulting from the COVID-19 pandemic.

However, the abrupt changes resulting from the pandemic highlight how changes such as virtual meetings and complete overhauls of clinic grids are possible when a mandate for change is present. As hospital systems grapple with the financial consequences of the COVID-19 pandemic, leadership will need to be mindful not to create additional inflexibilities in faculty schedules. This is especially pressing right now as faculty grapple with the complex challenges related to limited childcare options and virtual or remote learning for their children.

The responses from our survey also illustrate the myriad of “microinequities”^26^ experienced by physician parents. These individual inequities can additively create larger burdens^27^ that contribute to the stalled career progression for women. Current data reflect that only 21% of full professors and 15% of department chairs are women,^28^ and this work further illustrates how the lack of gender parity is not simply a pipeline issue or “glass ceiling” effect but also one of a “sticky floor” that adds additional challenges to women juggling parenting responsibilities. At current rates, gender parity will not be achieved for 50 years.^29^ To improve this timeline, effective approaches need to be integrated with engaged senior leadership.

A strength and contribution of our study is that we were able to elicit physician narratives from multiple specialties, with a high volume of respondents providing narrative comments—especially for a qualitative analysis with greater than 200 responses. This volume and representation across specialties enabled us to determine opportunities for improvement at the systems level. Addressing these opportunities and challenges will be necessary to improve institutional culture around parenting, and to ultimately increase gender parity in academic medicine.

Additional strengths of our study include sample selection and survey methods that optimally captured diverse experiences and perceptions to enhance generalizability. Collecting data through an electronic survey as opposed to structured verbal interviews as done in similar qualitative studies^21,25^ and sampling all physician faculty at a large academic health system encouraged a large sample size, minimized researcher-driven sampling bias, and included broader demographic representation of factors that may potentially influence the experience of parenting as a physician, such as years of experience as a faculty member, number of children, partner status, partner's work status, specialty, and full-time equivalent status.

Limitations of our work include that it is a single-center study, the response rate for the qualitative portion introduces possible nonresponse bias, and the majority of free-text comments were from physician parents. These limitations potentially impact the generalizability of our findings; however, we believe that there is enough in common with other academic medical institutions and that the themes and challenges put forth are universal enough to provide a valuable contribution to the ongoing dialog and work invested in physician work-life balance. The survey was also administrated before the COVID-19 pandemic. Future work will need to investigate how physician parents' experiences have been further challenged by the enormity of disruptions resulting from this pandemic.

## Conclusion

This study identified challenges for physician faculty at a large academic institution that convey the new reality for physician parents. To promote a supportive work environment that encourages career development and professional growth for physicians of all genders, these challenges need to be addressed, and novel solutions must be considered.

## Supplementary Material

Supplemental data

Supplemental data

## Supplementary Material

Supplementary Appendix SA1

Supplementary Appendix SA2
