# Supplementary material for: Operational Constraints and Gender Biases: A Qualitative Analysis of Physician Parenting Experiences
Source: Womens Health Rep (New Rochelle). 2022 Mar 4;3(1):297–306. doi: 10.1089/whr.2021.0099 (PMC8994438; doi:10.1089/whr.2021.0099)
Supplement: Supplemental data [file Suppl_AppendixSA1.docx]

**Survey**

1. Are you a parent
   1. Yes
   2. No
2. Please indicate the age of your child or children (check all that apply)
   1. <6
   2. 6-18
   3. >18
3. Have you ever been pregnant?
   1. Yes
   2. No
4. Have you been pregnant while working as a faculty member at the University of Michigan?
   1. Yes
   2. No
5. Who is your primary childcare during work hours? (or was your primary child care if you child(ren) are older) - Selected Choice
   1. Individual hired help (nanny, babysitter, etc)
   2. Daycare or other group facility
   3. Spouse
   4. Other family member
   5. None
   6. Other (free text)
6. Who is your primary childcare during work hours? (or was your primary child care if you child(ren) are older) - Other: - Text
7. Are you or your partner planning a pregnancy in the next 5 years?
   1. Yes
   2. No
8. What resources would be helpful to you as you are thinking about pregnancy? - Selected Choice
   1. Departmental/University Policies on Scheduling Personal Medical Appointments
   2. Assistance with selection of Obstetrical and/or CNM provider
   3. University of Michigan policy on parental leave
   4. University of Michigan Health Plan Fertility Treatment Coverage
   5. General Information Regarding Fertility Treatment/Preservation
   6. Other:
9. What resources would be helpful to you as you are thinking about pregnancy? - Other: - Text
10. Do you feel adequately knowledgeable regarding the University of Michigan policy on parental leave (including maternal, paternal, and adoption)?
    1. Definitely yes
    2. Probably yes
    3. Might or might not
    4. Probably not
    5. Definitely not
11. Do you feel adequately knowledgeable regarding your departmental policy and practices on parental leave (including maternal, paternal or adoption?)
    1. Definitely yes
    2. Probably yes
    3. Might or might not
    4. Probably not
    5. Definitely not
12. Do you feel adequately knowledgeable regarding Michigan Medicine resources for lactation support?
    1. Probably yes
    2. Might or might not
    3. Probably not
    4. Definitely not
13. My department leadership is supportive of faculty members during pregnancy
    1. Strongly agree
    2. Somewhat agree
    3. Neither agree nor disagree
    4. Somewhat disagree
    5. Strongly disagree
14. My department is accommodating of schedule flexibility related to pregnancy
    1. Strongly agree
    2. Somewhat agree
    3. Neither agree nor disagree
    4. Somewhat disagree
    5. Strongly disagree
15. There is a negative stigma attached to being pregnant while working as a faculty member
    1. Strongly agree
    2. Somewhat agree
    3. Neither agree nor disagree
    4. Somewhat disagree
    5. Strongly disagree
16. Male physicians who have children fall behind in the promotions process
    1. Strongly agree
    2. Somewhat agree
    3. Neither agree nor disagree
    4. Somewhat disagree
    5. Strongly disagree
17. Female physicians who have children fall behind in the promotions process
    1. Strongly agree
    2. Somewhat agree
    3. Neither agree nor disagree
    4. Somewhat disagree
    5. Strongly disagree
18. I feel comfortable discussing work-parenting-integration issues with my division leadership
    1. Strongly agree
    2. Somewhat agree
    3. Neither agree nor disagree
    4. Somewhat disagree
    5. Strongly disagree
19. I feel comfortable discussing work-parenting-integration issues with my department chair
    1. Strongly agree
    2. Somewhat agree
    3. Neither agree nor disagree
    4. Somewhat disagree
    5. Strongly disagree
20. Because of parenting or parenting commitments, I have: (select all that apply) - Selected Choice
    1. Turned down a project at my institution
    2. Turned down a leadership role at my institution
    3. Turned down a position at my institution
    4. Not participated in an institutional or departmental committee
    5. Not presented at a national meeting
    6. Felt compelled to take on a project
    7. Felt compelled to take on a leadership role at my institution
    8. Felt more efficient during my work day
    9. Other
    10. None of the above
21. Because of parenting or parenting commitments, I have: (select all that apply) - Other – Text
22. Is there anything you wish to share about your experience of pregnancy or parenting as a physician? (free text)
23. I wish I had more guidance on local resources about the following categories before and/or during pregnancy: (check all that apply) - Selected Choice
    1. Flexible Work Schedules
    2. Lactation Facilities
    3. Pet Care
    4. Meal Planning
    5. Reproductive, Endocrine and Infertility Services
    6. Unpredictable or emergency childcare options (eg. illness or snow days)
    7. Childcare
    8. Parental Leave
    9. Grocery Delivery
    10. Kid Friendly Activities in Ann Arbor
    11. Cleaning Services
    12. Dry Cleaning
    13. Other
24. I wish I had more guidance on local resources about the following categories before and/or during pregnancy: (check all that apply) - Other – Text
25. How early would you recommend establishing childcare plans (i.e. enroll in day care, hire a nanny, etc.)?
    1. Before pregnancy
    2. First trimester
    3. Second trimester
    4. Third trimester
26. What local services, day cares, or childcare options would you recommend to future physician parents? (free text)
27. What year did you complete your postgraduate training? (free text)
28. What year did you begin working at the University of Michigan as a faculty member? (free text)
29. What department do you currently work in?
    1. Obstetrics & Gynecology
    2. Anesthesiology
    3. Family Medicine
    4. Pediatrics – General
    5. Pediatrics - Hospital Medicine
    6. Pediatrics - Pulmonology
    7. Pediatrics - Neonatal-Perinatal Medicine
    8. Pediatrics - Developmental Behavioral
    9. Pediatrics – Neurology
    10. Pediatrics – Cardiology
    11. Pediatrics - Gastroenterology
    12. Pediatrics - Adolescent Medicine
    13. Pediatrics - Hematology/Oncology
    14. Pediatrics - Infectious Diseases
    15. Pediatrics - Critical Care Medicine
    16. Pediatrics - Genetics, Metabolism & Genomic Medicine
    17. Internal Medicine - Hospital Medicine
    18. Internal Medicine - General Medicine
    19. Internal Medicine – Rheumatology
    20. Internal Medicine - Infectious Diseases
    21. Internal Medicine - Hematology and Oncology
    22. Internal Medicine - Pulmonary and Critical Care
    23. Internal Medicine – Nephrology
    24. Internal Medicine - Gastroenterology and Hepatology
    25. Internal Medicine - Metabolism, Endocrinology & Diabetes
    26. Internal Medicine - Geriatric and Palliative Medicine
    27. Internal Medicine - Cardiovascular Medicine
    28. Internal Medicine - Allergy and Clinical Immunology
    29. Orthopaedic Surgery
    30. Physical Medicine and Rehabilitation
    31. Neurology
    32. General Surgery
    33. Surgery
    34. Psychiatry
    35. Emergency Medicine
    36. Radiation Oncology
    37. Radiology
    38. Pathology
    39. Ophthalmology & Visual Sciences
    40. Cardiac Surgery
    41. Urology
    42. Otolaryngology - Head and Neck Surgery
    43. Neurosurgery
    44. Dermatology
    45. Vascular Surgery
    46. Thoracic Surgery
30. What is your gender identity? - Selected Choice
    1. Male
    2. Female
31. What is your gender identity? - Other - Text
