# Supplementary material for: Operational Constraints and Gender Biases: A Qualitative Analysis of Physician Parenting Experiences
Source: Womens Health Rep (New Rochelle). 2022 Mar 4;3(1):297–306. doi: 10.1089/whr.2021.0099 (PMC8994438; doi:10.1089/whr.2021.0099)
Supplement: Supplemental data [file Suppl_AppendixSA2.docx]

Dear faculty,

There are many factors that affect the experiences of physician faculty at Michigan Medicine. We are interested in learning more about how one of these factors, the role of parenting, currently contributes to or has contributed to the faculty experience. We also welcome responses from faculty planning to start a family.  We hope to use this information to identify ways that we can be supportive for all of our faculty. Please complete this brief anonymous survey that should take no more than 5 minutes of your time.

<https://umich.qualtrics.com/jfe/form/SV_3DY09P9zwl1WaPz>

Once you send out the survey, then we will plan on sending weekly reminders for three additional weeks, then will close out the survey (and the email list will be eliminated as well.)

We are looking forward to the collection of this important information, and thank you again.

Sincerely,

Carol R. Bradford, MD, MS, FACS

Executive Vice Dean for Academic Affairs, Medical School

Chief Academic Officer, Michigan Medicine

Professor, Otolaryngology - Head & Neck Surgery

Helen Kang Morgan MD

Associate Professor, Obstetrics and Gynecology and Learning Health Sciences

Heather Burrows MD, PhD

Associate Chair of Education, Department of Pediatrics

Durga Singer M.A., M.D.

Assistant Professor of Pediatrics

Faculty Lead in Faculty Development

Lead for Gender DE&I activities in the Department of Pediatrics

Kirk Brower MD

Faculty Director, Michigan Medicine Wellness Office and Professor of Psychiatry
